# Genome Sequence of *Aeribacillus pallidus* Strain GS3372, an Endospore-Forming Bacterium Isolated in a Deep Geothermal Reservoir

**DOI:** 10.1128/genomeA.00981-15

**Published:** 2015-08-27

**Authors:** Sevasti Filippidou, Marion Jaussi, Thomas Junier, Tina Wunderlin, Nicole Jeanneret, Simona Regenspurg, Po-E Li, Chien-Chi Lo, Shannon Johnson, Kim McMurry, Cheryl D. Gleasner, Momchilo Vuyisich, Patrick S. Chain, Pilar Junier

**Affiliations:** a Laboratory of Microbiology, Neuchatel, Switzerland; b Vital-IT Group, Swiss Institute of Bioinformatics, Lausanne, Switzerland; c German Research Centre for Geosciences GFZ, Potsdam, Germany; d Los Alamos National Laboratory, Los Alamos, New Mexico, USA

## Abstract

The genome of strain GS3372 is the first publicly available strain of *Aeribacillus pallidus*. This endospore-forming thermophilic strain was isolated from a deep geothermal reservoir. The availability of this genome can contribute to the clarification of the taxonomy of the closely related *Anoxybacillus*, *Geobacillus*, and *Aeribacillus* genera.

## GENOME ANNOUNCEMENT

*Aeribacillus pallidus* is the only species of the novel *Aeribacillus* genus (1). This species was previously classified as *Bacillus pallidus* (2) and later as *Geobacillus pallidus* (3). The type strain (strain H12) was isolated from thermally treated sewage, and its full genome is not publicly available. More strains of this species have been isolated from hot springs (4, 5), production water from an oil reservoir (6), and crude-contaminated soil (7). Strain GS3372 was isolated multiple times from fluid samples (50 to 70°C) from the deep geothermal reservoir of Gross Schoenebeck, in the North German Basin (52°54′13.15″ N, 13°36′5.43″ E). Analysis of the 16S rRNA gene sequence (99% identity) and DNA-DNA hybridization (79.5% similarity) indicated that GS3372 is a novel strain of *A. pallidus*.

Genomic DNA was extracted from an overnight culture using the QIAamp DNA minikit (Qiagen GmbH, Germany). For this genome, an Illumina short-insert paired-end library was constructed and sequenced, which generated 4.744 Mbp of data, of which 2.41 Mbp is included in the final assembly (482× genome coverage) (8). The data were assembled with Velvet, version 1.2.08 (9), to an estimated size of 4.9 Mbp with a 57.4% G+C content. Genome annotation utilized an Ergatis-based (10) workflow with minor manual curation then visualized with the Artemis genome browser and annotation tool (11). The complete genome sequence contained 5,015 genes, 9 rRNAs (5S, 16S, and 23S), 69 tRNAs, and 4 noncoding RNAs (ncRNAs) predicted. The proteome of GS3372 revealed the presence of 96 genes related to the sporulation and germination pathways.

The GS3372 genome contains copper oxidase and manganese catalase genes, as well as genes encoding proteins related to arsenic (ArsB) and copper (CopZ) resistance. While the *A. pallidus* type strain is nonmotile, strain GS3372 is motile, and accordingly, the loci *flhA* and *flgG* related to flagellar synthesis are present in its genome. Moreover, the ability of this strain to assimilate a large number of carbon sources is depicted in the genome by the presence of transporter, permease, and isomerase genes for glucose, d-xylose, glycerol, ribose, and mannose, all of which are supported by biochemical characterization (S. Filippidou and P. Junier, unpublished data). Finally, YflT which is related to heat-induced thermotolerance (12), is found in its proteome. These observations act in accordance with the ecology of the strain, which was isolated in a thermophilic and oligotrophic environment.

### Nucleotide sequence accession numbers.

This whole-genome shotgun project has been deposited at DDBJ/EMBL/GenBank under the accession no. JYCD00000000. The version described here is JYCD00000000.1.
